# Case of Poisoning with Arsenic

**Published:** 1848-01

**Authors:** 


*Case of Poisoning with Arsenic.* [The details of the following case will doubtless create surprise in the minds of many. The poison was taken on Wednesday, and at 10 o'clock at night the vomiting commenced. On Thursday morning M. Louis saw him, but did not recognise the symptoms of poisoning. On Friday, Andral was called in, but he also failed to detect the symptoms of poisoning. On Saturday evening, *three days after the poison had been taken*, the symptoms were for the first time recognized; and on Sunday morning, M. Chevalier detected arsenic. Truly the Parisian *Savans* are not immaculate.—The following is an extract from the Chancellor's report to the Court of Peers.—*London Lancet.*]

"Finding himself overwhelmed by the appearances of a conviction from which he could not escape, he immediately, without doubt, adopted the determination to use the poison with which he was provided. He thought, according to all appearance, that the effect would be more prompt than it was. He certainly took the poison in the course of the Wednesday, either a little earlier or a little later, but in such a way that the evacuations which it would produce, considering the dose that he had swallowed, began at ten at night in a way to excite serious attention. The rest of the night and the following day were very bad. The evacuations had ceased at the end of Thursday, but were succeeded by great weakness. A physician of great ability, who had attended him and his family, was sent for, but did not arrive till the morning of Thursday. Notwithstanding the inquiries he entered into, and which appeared to be very serious, he did not recognize the symptoms of poison, but thought he discovered in the patient those of an attack of cholera. He ordered the remedies usually prescribed in such cases. The rest of that day and the following night were past most painfully, but in the morning of Friday his condition seemed to be ameliorated. Having already invested himself with the power conferred by the royal ordinance rendered the day before, and which had just reached me, I thought myself bound about the middle of Friday to call in Dr. Andral officially to examine most profoundly the state of M. de Praslin. Dr. Andral came immediately to him and examined him with the greatest care, but from the improvement which had taken place he was not able to discover the true cause of the evil. At my desire he went again to the bedside of the patient, and on his return assured me that his removal to the prison of the Luxembourg might be made without inconvenience. It took place, in fact, on Saturday, at five in the morning, and, in the course of the day, I was able to interrogate him in the presence of the peers who had accepted the charge of aiding me in so laborious a task. That examination will be found in the papers before you. Although a complete avowal did not, during the whole length of it, pass the lips of the accused, the absence of all former denegation, even when the option between a yes and a no was formally given him, might well pass for an avowal. This examination could not continue any length of time, the state of weakness into which the accused had fallen did not permit it to be prolonged. He was immediately carried back to his bed, which he did not afterwards quit. The same evening, the symptoms became infinitely more grave, and all the appearance of poisoning became more evident. From that time he was treated as was proper in such a case by M. Andral, M. Rouget, the physician of the Luxembourg, and M. Louis, his usual medical attendant, it was the latter who saw him from the Thursday. On Sunday morning I ordered medical experiments to be made on the dejections of the accused of every kind M. Chevalier, who was charged with the operation, perceived very clearly the presence of arsenic in the matter submitted to him. He also discovered the presence of arsenic in a phial seized by M. de Praslin, whilst still in his hotel, and which probably contained that which he had taken. Afterwards he ascertained the presence of arsenic in the dejections which remained on an arm-chair, where M. de Praslin had been placed on the Thursday on getting out of a bath, and which had been removed to the garden in consequence of the stench proceeding from it. From Sunday morning, the aggravation of the illness, the alternatives of pain and weakness which it caused, did not permit a new examination of M. de Praslin to be attempted. Only some detached phrases were drawn from him, but it was impossible to subject him to a regular examination, attempted at once, but without success. His death took place on Tuesday, and the autopsy of the body was effected by Messrs. Orfila and Tardieu, in the presence of Drs. Andral, Louis, and Rouget. The proceedings and the experiments which were afterwards instituted by Messrs. Orfila and Tardieu, fully confirmed the assertion of M. Chevalier. The conclusions come to, tend to show that the poisoning of M. de Praslin, effected by himself, in the middle of the day, some hours only after the murder had been perpetrated. The *proces verbaux*, which are before you, show that all the incidents which took place afterwards, the intervals which elapsed between them, in fine, the duration of the state which terminated in death, are the natural and usual consequences of this kind of poisoning."

